# Amplification of the poorer ear by StereoBiCROS in case of asymmetric sensorineural hearing loss: effect on tinnitus

**DOI:** 10.3389/fnins.2023.1141096

**Published:** 2023-05-25

**Authors:** Morgan Potier, Stéphane Gallego, Philippe Fournier, Mathieu Marx, Arnaud Noreña

**Affiliations:** ^1^CNRS UMR, Laboratoire de Neurosciences Cognitives, Aix-Marseille–Université Centre Saint-Charles, Marseille, France; ^2^Laboratoire d’Audiologie Clinique, Narbonne, France; ^3^Institut des Sciences et Technologies de Réadaptation, Université de Lyon, Lyon, France; ^4^Département de réadaptation, Faculté de Médecine, Université Laval, Quebec, QC, Canada; ^5^CNRS UMR, Centre de Recherche Cerveau et Cognition (CERCO), Université Paul Sabatier, Toulouse, France

**Keywords:** asymmetric hearing loss, Single-Sided Deafness, tinnitus, hearing aid, Contralateral Routing of Signal, StereoBiCROS, TriCROS, cochlear implant

## Abstract

Tinnitus is prevalent among patients suffering from Single-Sided Deafness (SSD) and Asymmetrical Hearing Loss (AHL). In addition to bothersome tinnitus in the poorer ear, these patients also report issues with understanding speech in noise and sound localization. The conventional treatment options offered to these patients to improve auditory abilities are cochlear implantation, bone conduction devices or Contralateral Routing Of Signal (CROS) hearing aids. It was recently found that the benefit of cochlear implantation for tinnitus associated with AHL/SSD was greater than the other two approaches. It is conceivable that the lack of stimulation provided to the poorer ear in these last approaches explains their modest impact on tinnitus perception. A new technology that combines the ability to reroute the sound from the poorer ear to the good ear (CROS system) while still stimulating the poorer ear with conventional sound amplification has recently been developed: the StereoBiCROS system. The aim of this study was to investigate the effects of this new device on tinnitus. Twelve AHL and two SSD patients aged 70.7 ± 7.9 years with tinnitus were fitted with bilateral hearing aids that included 3 programs: Stereophonic, BiCROS and StereoBiCROS (CROS + bilateral amplification). The short-and long-term effect of the approach on tinnitus was assessed using a tinnitus Loudness Visual Analog Scale (VAS) and the Tinnitus Handicap Inventory (THI), respectively. Both the VAS and the THI were used before and one month after the hearing aid fitting. Of the 14 patients who used their hearing aids daily (12.6 ± 1.6 h per day) the StereoBiCROS program was the most used program (81.8 ± 20.5% of the time). The average THI total score decreased from 47 (± 22) to 15 (± 16) (*p* = 0.002) and the VAS-Loudness score decreased from 7 (± 1) to 2 (± 2) (*p* < 0.001) after the one-month trial period. In conclusion, StereoBiCROS stimulation strategy seems to offer an effective alternative to reduce tinnitus handicap and loudness for patients with AHL/SSD and tinnitus. This effect may be driven by sound amplification of the poorer ear.

## Introduction

In the field of hearing restoration, the treatment of severe-to-profound unilateral hearing loss is always challenging, both in the case of pure Single-Sided Deafness (SSD) and bilateral Asymmetric Hearing Loss (AHL) because these hearing profiles correspond to heterogeneous hearing disorders (Vila and Lieu, 2015; Usami et al., 2017). SSD is defined as a Pure Tone thresholds Average (PTA) over 0.5, 1, 2 and 4 kHz exceeding 70 dB HL in the poorer ear, and better than 30 dB HL in the good ear. In AHL, PTA also exceeds 70 dB HL in the poorer ear and is between 30 dB HL and 55 dB HL in the better ear (Usami et al., 2017). These types of hearing loss are accompanied by dramatic functional consequences. The loss of binaural cues, i.e., interaural time and level differences, impairs spatial localization (Chan et al., 2008; McLeod et al., 2008; Vermeire and Van de Heyning, 2009; Buechner et al., 2010; Arndt et al., 2011; Jacob et al., 2011; Agterberg et al., 2014) and speech understanding in noisy environments (Sargent et al., 2001; Welsh et al., 2004; Bishop and Eby, 2010; Avan et al., 2015). Moreover, tinnitus is often reported (in 54 to 84% of cases) in the poorer ear (Quaranta et al., 2004; Wie et al., 2010) and is even considered more troublesome than the hearing difficulties by some AHL patients (Chiossoine-Kerdel et al., 2000; Zöger et al., 2001; Van de Heyning et al., 2011; Bhatt et al., 2016). Tinnitus has recently been defined as an auditory sensation without an external sound stimulation or meaning, which can be lived as an unpleasant experience, possibly impacting the quality of life (Noreña et al., 2021). Tinnitus can lead to severe psychological comorbidities such as depression and anxiety (Zöger et al., 2001; Van de Heyning et al., 2011; Bhatt et al., 2016). Currently, there is no well-established medical cure for tinnitus (Vio and Holme, 2005) and clinical management options consist of developing coping strategies to better manage the impact of tinnitus on daily living (Jastreboff, 1999; Cima et al., 2012).

Subjective tinnitus has been suggested to result from the central changes due to hearing loss and the sensory deprivation that is associated to it (Noreña, 2011, 2015; Eggermont and Roberts, 2012; Roberts et al., 2013). In this context, restoring at least part of the sensory inputs lost after cochlear damages with a Hearing Aid (HA) or using electric stimulation delivered by a Cochlear Implant (CI) may reverse the tinnitus-related central changes and decrease or suppress the tinnitus percept (Noreña and Eggermont, 2005; Noreña, 2011; Eggermont, 2015). The acoustic/electric stimulation may also reduce the tinnitus impact by masking tinnitus and/or triggering extended periods of time without tinnitus (residual inhibition) (Hoare et al., 2014a,b; Sereda et al., 2018). In the context of SSD/AHL with tinnitus, the implementation of CI’s has progressively increased over the years, from the pioneer study by Van de Heyning et al. (2008) to more studies worldwide (Kleinjung et al., 2009; Buechner et al., 2010; Carlyon et al., 2010; Arndt et al., 2011; Punte et al., 2011; Arts et al., 2012; Firszt et al., 2012; Blasco and Redleaf, 2014; Gartrell et al., 2014; Vlastarakos et al., 2014; Van Zon et al., 2015; Peter et al., 2019; Levy et al., 2020). Using CIs is an approach to treat tinnitus in AHL/SSD cases has recently been approved by the French National Authorities for Health (Official Journal of September 8, 2021 n°0209). The indications for CI should evolve because some studies have shown reduction rates in tinnitus distress in 77 to 100% in SSD patients (Punte et al., 2011; Van Zon et al., 2015) and a recent systematic review showed that CI improved tinnitus handicap was observed in 87.9% of cases (with 34.2% reporting complete abolition), stability in 7.3%, while worsening was reported in only 4.9% of cases (Peter et al., 2019). In brief, there is a good level of evidence showing that unilateral CI in SSD patients can improve the tinnitus severity (Levy et al., 2020). These results are strongly consistent with the effects of CI on tinnitus in patients with bilateral deafness (Baguley and Atlas, 2007; Andersson et al., 2009; Pan et al., 2009; Amoodi et al., 2011; Bovo et al., 2011; Olze et al., 2011; Kompis et al., 2012; Kim et al., 2013, 2016; Ramakers et al., 2015).

While cochlear implantation is reasonably efficient to reduce tinnitus in some patients, it comes however with serious disadvantages: the cochlear implantation may further destroy any residual hearing or vestibular function in the impaired ear, as any surgery comprises surgical risks, and the financial cost is relatively high (Venail et al., 2008; Farinetti et al., 2014; McKinnon, 2014; Theunisse et al., 2018; Marx et al., 2019). In the context of SSD with residual hearing or AHL, alternative strategies to CI can be used to restore hearing. When the better ear is sub-normal like in the case of SSD, Contralateral Routing Of Signal HA (CROS) can be used (Harford and Barry, 1965). The CROS rerouting the acoustic signal captured on the side of the poorer ear toward the contralateral normal ear. For AHL, a BiCROS system is preferred (Harford, 1966; Harford and Dodds, 1974). Like the CROS system, it allows the reroutes of the acoustic signal from the poorer ear to the better ear. In addition, it adds sound amplification in the hearing-impaired better ear (Harford and Barry, 1965; Harford, 1966; Harford and Dodds, 1966). Historically, binaural amplification in the case of AHL, that is amplification of both the better and the poorer ear, has been discarded due to the possible occurrence of a phenomenon known as binaural interference, a condition in which the response to the poorer ear interferes with the response of the better ear (Jerger et al., 1993). In the four cases presented in the Jerger study, participants with bilateral hearing impairment performed better at a speech perception test with one HA fitted in the better ear than when both HAs been fitted. They also found altered auditory middle latency amplitudes for the binaural condition than for the monaural conditions in some subjects. However, this effect may be overcome following the long-term use of amplification (Bishop et al., 2017). In addition, the specific impact of amplification of the poorer ear on the tinnitus of AHL patients has not been tested experimentally. Finally, the rerouting principle may also be performed using bone conduction devices, whether implanted or not, which have shown some benefits in certain spatial configurations for speech in noise recognition (Baguley et al., 2006; Hol et al., 2010; Desmet et al., 2012; Snapp et al., 2017; Picou et al., 2020). However, as the same bone conduction sound signal is transferred to both inner ears simultaneously, it is usually adjusted for the hearing loss profile of the better ear thus providing less stimulation to the poorer ear. This may partially explain why bone conduction systems have shown less benefit on tinnitus than CI in SSD and AHL cases (Marx et al., 2021).

Recently, a system that combines the benefits offered by a BiCROS system with the amplification of the poorer ear was developed by the HAs manufacturers: the StereoBiCROS (or named TriCROS by some audiologists). To the best of our knowledge, no study investigated the effect of the amplification of the poorer ear on the tinnitus intensity and handicap for AHL patients, more so, by using a StereoBiCROS stimulation. The aim of the present study was to investigate the effect on tinnitus intensity and burden of amplification of the poorer ear on patients with AHL/SSD using StereoBiCROS HA stimulation. We hypothesized that providing amplification to the poorer ear in addition to the better ear will reduce tinnitus intensity and handicap.

## Materials and methods

### Study design

The study is a one group pretest posttest quasi-experimental design.

### Patients

Twelve AHL and two SSD patients with tinnitus were recruited among 18 patients with SSD or AHL who were initially tested for another study evaluating speech perception and localization using the StereoBiCROS. For the AHL cases, the poorer ear was localized on the left side for 7 cases and the right side for 5 cases. The poorer ear was localized only on the left side for the SSD cases. The etiologies of the SSD/AHL were sudden sensorineural hearing loss for nine patients, sound trauma for 4 patients, and chronic otitis media for one patient. The inclusion criteria included being over 18 years old and being a native French speaker with AHL/SSD diagnosed more than 6 months ago. In addition, only patients with chronic subjective tinnitus (more than 6 months) and who had never worn HA (stereophonic or BiCROS) were included in this study. Prior tinnitus intervention was not an exclusion criterion. Patients with fluctuating hearing loss and/or objective, pulsatile or somatosensory tinnitus were excluded from this study. Participants were informed that they could not follow other tinnitus interventions while participating in this study. Participants with conductive hearing loss were also excluded from this study. Patients were considered with objective tinnitus if they reported during the case history pulsatile and/or rhythmic tinnitus. They were considered with somatosensory tinnitus if they reported any modulation of their tinnitus following head, neck or jaw movement. Seven women and seven men aged between 54 to 83 years old (mean age = 70.7 y.o.) participated in the study. They all reported continuous tinnitus, with 11 participants reporting unilateral tinnitus localized in the poorer ear and 3 with bilateral tinnitus localized in both ears. Ten out of the 14 patients experienced significant tinnitus disability as defined by a score higher than 38 on the Tinnitus Handicap Inventory questionnaire (THI) (Newman et al., 1996). The tinnitus was described as a whistle sound (*n* = 7), a buzzing sound (*n* = 4), a swish sound (*n* = 2) or a wind-like sound (*n* = 1). The descriptive information’s including the duration of the deafness, the poorer side, the pure tone thresholds averaged for the poorer and the better ear and the specific details about tinnitus (laterality, description, and bothersome character) are summarized in Table 1. This study was carried out in accordance with the Declaration of World Medical Association (2013) and the protocol was approved on 05/23/2019 by the West 6 Personal Protection Committee (CPP N ° 11,555-DM2). All participants volunteered and provided written informed consent before their participation in the study.

**Table 1:** Patients’ characteristics.

# Patient	Gender	Age (years)	Etiology	Deafness duration (months)	SSD/ AHL	Poorer ear	Tinnitus laterality	Tinnitus description	Bothersome tinnitus	Better ear PTA (dB HL)	Poorer ear PTA (dB HL)	Better ear amplified PTA (dB HL)	Poorer ear amplified PTA (dB HL)
1	Male	69	Sound trauma	362	SSD	L	R/L	Whistle	N	28	102	28	58
2	Female	62	Sudden deafness	48	AHL	L	L	Swish	Y	34	90	18	64
3	Male	68	Sudden deafness	118	AHL	R	R	Swish	Y	34	103	16	65
4	Female	66	Sudden deafness	66	AHL	L	R/L	Whistle	Y	47	89	21	51
5	Male	71	Sudden deafness	109	AHL	L	L	Wind	Y	30	85	15	49
6	Male	70	Work in noise	-	SSD	L	L	Whistle	Y	24	70	14	41
7	Female	73	Sudden deafness	43	AHL	L	L	Whistle	Y	62	97	36	58
8	Male	80	ENT history	72	AHL	R	R	Buzzing	N	55	70	29	38
9	Female	80	Sudden deafness	56	AHL	L	R/L	Buzzing	N	44	88	19	53
10	Male	78	Work in noise	–	AHL	R	R	Whistle	Y	48	94	20	61
11	Female	83	Sudden deafness	286	AHL	L	L	Buzzing	Y	52	102	29	54
12	Male	71	Work in noise	–	AHL	R	R	Buzzing	N	42	69	26	50
13	Female	65	Sudden deafness	40	AHL	R	R	Whistle	Y	35	77	16	44
14	Female	54	Sudden deafness	91	AHL	L	L	Whistle	Y	52	79	24	46
Mean (SD)		70.7 (7.9)		117.4 (106.7)						41.9 (11.4)	86.8 (12.2)	22.1 (6.6)	52.1 (8.3)

PTA, Pure Tone thresholds (in dB HL) averaged at frequencies 500, 1,000, 2,000, and 4,000 Hz. R = Unilateral Right, L = Unilateral Left, R/L = Bilateral, Y = Yes, N = No, − = no response. According to the Newman classification (Newman et al., 1998), bothersome tinnitus is considered when THI score > 38 and non-bothersome when THI score < 38. Individual PTAs are rounded up to the excess, average PTA is given at its exact value. The deafness duration is the time difference (in months) between the diagnostic and the speech recognition evaluation.

### Hearing evaluation

Audiological assessments were carried out in a double-walled soundproof room (ISO 6189 compliant). After otoscopy, a conventional hearing threshold test using TDH39 headphones calibrated in compliance with the ISO 8253 standard was performed. A tonal audiometry by air conduction (AC) at frequencies 0.25–8 kHz and bone conduction (BC) at frequencies 0.5–4 kHz were performed (B71 - Radioear Corporation, Pennsylvania, USA). The hearing thresholds were measured with warbled pure tones, following the Hughson and Westlake manual method (Hughson and Westlake, 1944). If a 10-dB HL gap was found between AC and BC thresholds at any frequency, the patient was excluded from the study. The thresholds exceeding the audiometer evaluation limits were settled at 120 dB HL. Speech audiometry was then performed in AC using the French Fournier’s dissyllabic word lists (Fournier, 1951), with a maximum intensity of 105 dB HL. The evaluation of the amplification gain, that is the hearing threshold with the activated hearing aids, was carried out in a free field for each ear. Stimuli were emitted by a loudspeaker positioned in front of the patient’s head, at one meter distance. To avoid standing waves, we used warble-tones for each tested frequency and the faintest sound they could hear, with the hearing aid on, was determined following the standard clinical procedure. The French Fournier’s dissyllabic word lists for speech audiometry were also presented in free field and the patient was asked to repeat the word they heard for each ear with the hearing aids on and without the hearing aids. For both free field hearing thresholds and speech audiometry, during the poorer ear testing, a narrow-band masking sound or a speech masking sound was applied to the better ear using TDH39 headphones to avoid cross-hearing. This masking technique ensured that we obtained the response from the poorer ear.

### HA fitting

**Figure 1:**
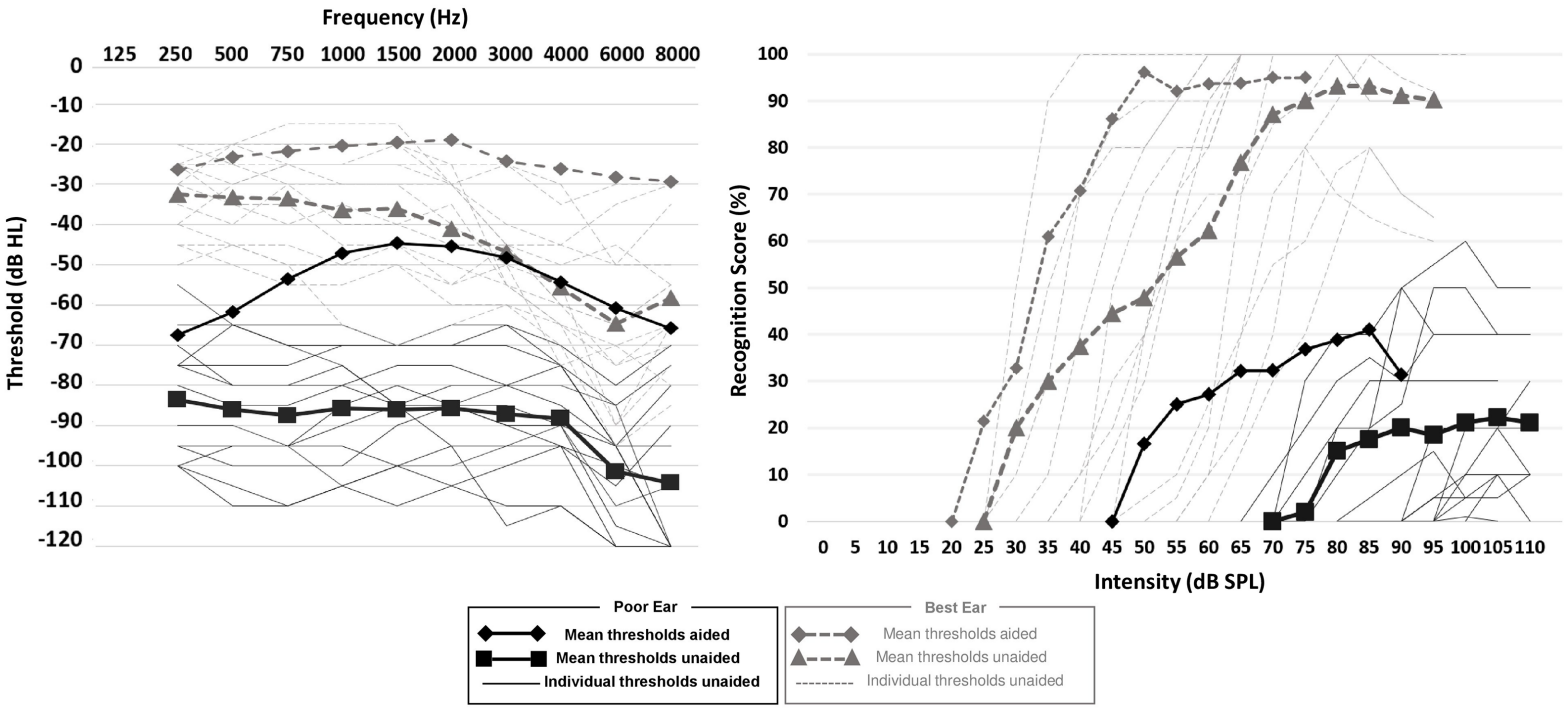
Hearing thresholds and speech audiometry. Continuous and dashed lines (without symbols) represent the individual unaided auditory thresholds for the poor and best ears, respectively. Black squares and diamonds represent the averaged unaided and averaged auditory thresholds in the poor ear, respectively. Gray triangles and diamonds represent the averaged unaided and aided auditory thresholds in the best ear, respectively.

Two HA models were used in this study: Sound SHD-9 and Audéo Belong-90 from Phonak hearing aid manufacturer (Sonova AG Stäfa, *Switzerland*). HA fitting was carried out by an experienced hearing instrument specialist (first author). Ear impression of each ear were taken to produce tailor-made earmolds to adapt, as much as possible, to external auditory canal and pinna anatomy. The optimal tightness was sought in the fitted ear (absent venting or 1 mm maximum venting diameter) to deliver maximum acoustic power output, while a more substantial venting (between 1 mm and 2.5 mm) was sought in the better ear, when needed. The BiCROS system allows a wireless transmission of the audio signal bandwidth between 130 Hz and 6.0 kHz from the transmitter device located on the poorer ear to the receiver device on the better ear. To achieve this, manufacturers used inductive transmission technology with digital coding of carrier frequency 10.6 MHz. HA fitting was performed using an Aurical Visible Speech system with a wireless SpeechLink 100 binaural measurement unit (Madsen, GN Otometrics, Taastrup, Denmark) to match specified amplification targets using NAL-NL2 methodology and Real Ear Measurement (REM). Figure 1 shows the averaged hearing thresholds and speech audiometry in both ears in the unaided and aided condition. The mean unaided thresholds are between 70-and 100-dB HL in the poorer ear dB HL (PTA = 86.8 ± 12.2 dB HL) and between 30-and 60-dB HL in the better ear (PTA = 41.9 ± 11.4 dB HL). The threshold improvement with the StereoBiCROS amplification was of 52.1 ± 8.3 dB in the poorer ear, and of 22.1 ± 6.6 dB in the better ear, on average. Regarding the speech audiometry, the mean maximum intelligibility was good in the better ear (90% on average in aided and unaided conditions), but very poor in the poorer ear (20% on average in the unaided condition and 40% in the aided condition).

During this wireless transmission, the microphone of the transmitting device was set in omni-directional mode. Three different programs were available to the patients: Stereophonic, BiCROS and StereoBiCROS. The Stereophonic program consisted of conventional bilateral amplification. The BiCROS and StereoBiCROS programs both consisted in rerouting signals coming from the poorer ear to the better ear. In addition, the BiCROS program included amplification only to the better ear side, the StereoBiCROS program also included amplification of the poorer ear (i.e., bilateral amplification). The patients could change the program at will by pressing a button on the HA device. The programs were ordered as follows, in a loop: Stereophonic, BiCROS and StereoBiCROS. See below for detailed instructions provided to patients.

### Subjective tinnitus assessment

To quantify tinnitus intensity and handicap during the one-month HA trial period, we used a Visual Analog Scale (VAS) and the French version of the Tinnitus Handicap Inventory (THI) questionnaire (Ghulyan-Bédikian et al., 2010), respectively. A VAS was used to assess the subjective tinnitus’ loudness (Miller and Ferris, 1993) as it was recommended by some authors as a way of measuring the effectiveness of a tinnitus treatment (De Ridder et al., 2010; Vanneste et al., 2011; Frank et al., 2012; Hall et al., 2017). The VAS consists of a 10 cm solid line labeled “no tinnitus” on one end and “highest tinnitus intensity” on the other. The patient is asked to place a mark along the line that best corresponds to their perception of tinnitus intensity (Wewers and Lowe, 1990).

The THI questionnaire is recommended by the scientific community (Langguth et al., 2007) to assess the handicap associated with tinnitus (Newman et al., 1998) and to evaluate any therapeutic effect of interventions (Westerberg et al., 1996; Mirz et al., 2000). The THI is psychometrically robust and reliable. It consists of 25 questions developed to cover three disability subscales: functional (12 questions), emotional (8 questions) and catastrophic (5 questions). For each question, three answers are possible: “yes” (scored 4 points), “sometimes” (scored 2 points), or “no” (scored 0 point). The total score is obtained by summing the scores for each question. The total score lies between 0 and 100. In addition, the total score can quantify the severity of tinnitus. The severity grades range from 1 to 5: no handicap (grade 1, scores between 0–16), mild (grade 2, scores between 18–36), moderate (grade 3, scores between 38–56), severe (grade 4, scores between 58–76) and catastrophic (grade 5, scores between 78–100), respectively. Following Newman classification, we considered a decrease of 20 points in the THI score as a clinically meaningful decrease (Newman et al., 1998).

### Procedure

The study took place at the « Laboratoire d’Audiologie Clinique » in Narbonne, France. At the first visit, the inclusion and exclusion criteria were assessed by a one-on-one interview with each potential candidate. Once the criteria were checked and the patient gave written consent to participate in the study, we proceeded with the audiological assessment including hearing thresholds and speech perception measurements. Afterwards, ear impression was taken on each side to produce tailor-made earmolds and the HAs were ordered from the supplier. A few days later, the patient was asked to come back to the clinic. The patient was asked to complete the THI questionnaire and to evaluate their tinnitus intensity using the VAS. After completing these measures, the patient was fitted with the HAs. The HAs were lent to the patients for the duration of the trial. Although the StereoBiCROS program was the default program activated when the HAs were turned on, patients were given instructions to test each of the three programs (Stereophonic, BiCROS, and StereoBiCROS) in different sound environments during the one-month trial period. They were instructed that they were free to move from one program to the other as many times as they want during the trial. At the end of the HAs fitting session, they were asked to complete the VAS of tinnitus intensity but this time with the HAs on while they were listening through the StereoBiCROS program. After the first 10 days of HAs trial, the patient was asked to come back to the clinic for a follow-up. This follow-up step was repeated 2 times in total (D + 10 and D + 20) over the 30-day trial period. During these sessions, minors’ amplification adjustments were performed by the hearing instrument specialist if the patient complained about discomfort. In addition, a datalogging protocol captured the use of the three different HA programs during the one-month trial period (D + 10, D + 20 and D + 30). At the end of the 30 days, the patient was seen at the clinic for the last time and was asked to complete the THI questionnaire and the tinnitus loudness VAS with the HAs off and then on.

### Statistical analysis

Statistical analyses were performed using the XLSTAT software (Addinsoft, New York, USA). The results of the multiple measurements are shown as the means ± standard deviation. The normality of the distribution was assessed by using the Kolmogorov–Smirnov test. Because normality was never reached for any of the variables tested, non-parametric statistical tests were used. To assess differences between the utilization time between each program, we performed the Pairwise Multiple Comparisons test (also called Nemenyi’s test). The repeated-samples two-sided Wilcoxon signed rank test and sign test analysis (Hollander and Wolfe, 1973) were used to test the putative differences between baseline and one month after HA fitting. Differences were considered as statistically significant when *p* < 0.05. To estimate the effect size in our population, we calculated Cohen’s dz. from the *t*-value and the number of participants using the formula provided by Rosenthal (1991).

## Results

### Effects on tinnitus assessed by the THI questionnaire

**Figure 2:**
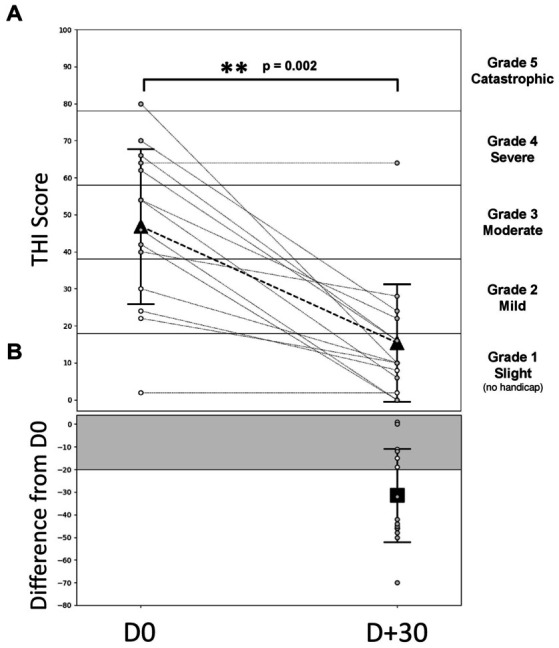
Evolution of the THI score during the follow-up. **(A)** Individual and average THI-score over time. Gray dashed lines represent individual patients, gray symbols for patients with THI score > 38 (bothersome tinnitus, *N* = 10) and white symbols for patients with THI score < 38 (non-bothersome tinnitus, *N* = 4). The triangle symbols represent the mean ± SD to D0 = free ear (before the HA activation) and for D + 30 (1 month after the HA wearing). The different areas indicate the different grades of THI: grade 1 (0–16), grade 2 (18–36), grade 3 (38–56), grade 4 (58–76), and grade 5 (78–100). **(B)** THI-score differences between D + 30 and D0. The square symbol represents the average THI difference (± SD) for all patients between D0 and D + 30. The gray area represents a decrease in 20 points which characterizes a significant decrease (Newman improvement criterion). *p*-values in the two-sided Wilcoxon signed rank test are obtained by comparing the values measured during D0.

The results provided in this section are the pooled results of the three HAs settings: since the patients were free to move from one program to the other during the entire trial period, any modification on the THI score would reflect the combination of all the three programs. The average THI scores decreased significantly between the baseline (D0: 46.9 ± 21.7) and the end of one month HA trial period (D + 30: 15.4 ± 16.5, Wilcoxon signed-test, *p* = 0.002, effect size of Cohen’s *dz* = 1.5). According to the Newman classification, 10 patients (71.4%) had moderate to severe tinnitus (THI score > 38) at the beginning of the trial (Figure 2A). If we consider a 20-point decrease in the THI score (Figure 2B, gray area) as a clinical meaningful decrease (Newman et al., 1998), 9 patients out of 14 (64%) displayed a clinically significant improvement in their tinnitus handicap score, 3 patients (21%) displayed a change that was not clinically significant, and 2 patients (15%) obtained the same score at D0 and D + 30. For the 3 patients with bilateral tinnitus, the THI scores between D0 and D + 30 decrease significantly less (10.7 decrease on average) compared with the score of the 11 patients reporting unilateral tinnitus (37.1 decrease on average).

### Effects on tinnitus assessed by the VAS-loudness

**Figure 3:**
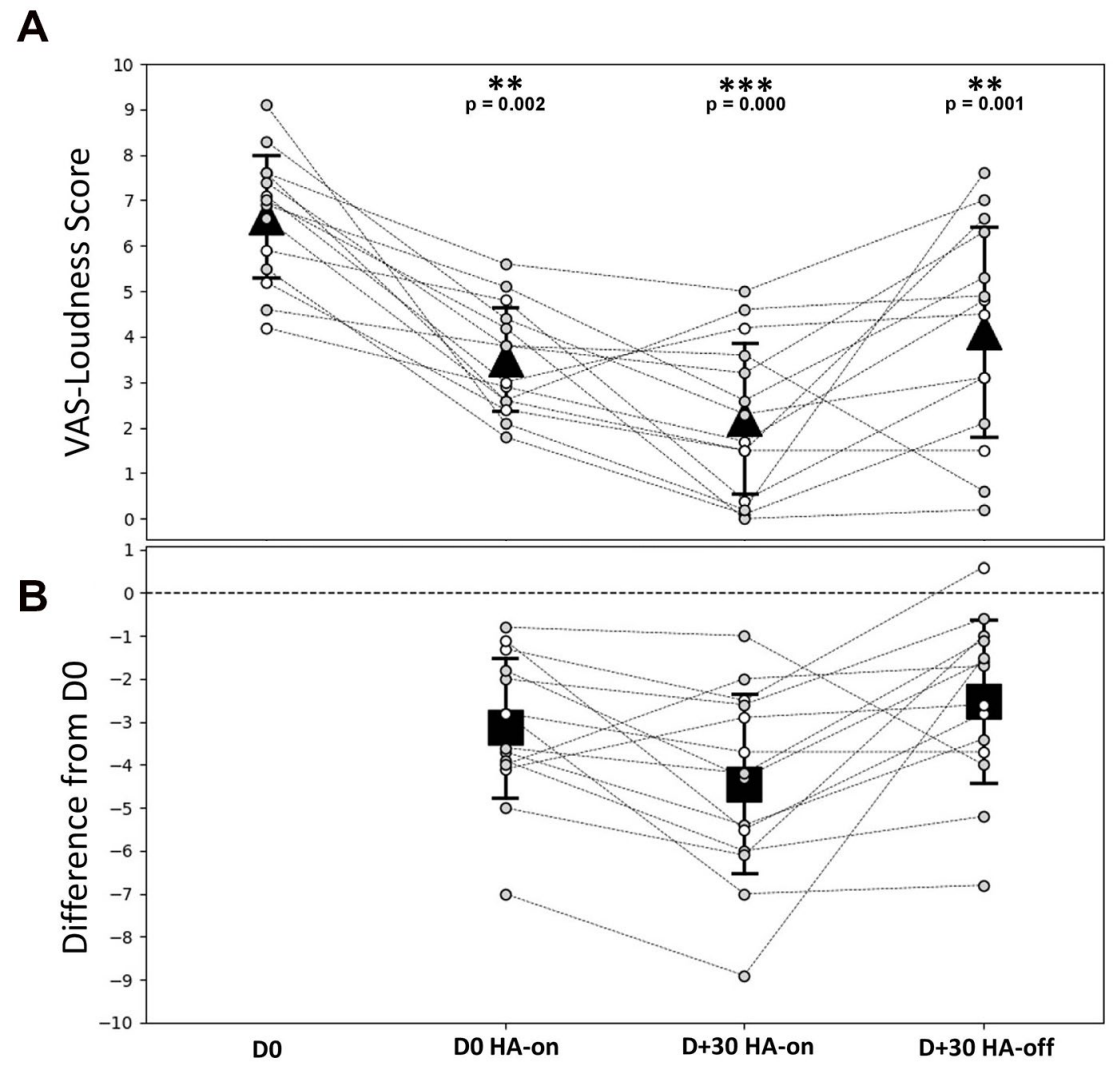
VAS-Loudness outcomes during follow-up. **(A)** Individual and average VAS-Loudness score for each time condition. **(B)** VAS-Loudness score differences (from D0) over time. Gray dashed lines represent individual patients, gray symbols for patients with bothersome tinnitus (THI score > 38, *N* = 10) and white symbols for patients with non-bothersome tinnitus (THI score < 38, *N* = 4). The triangle symbols represent the mean ± SD at different times during the follow-up. Statistical differences are indicated above the top panel. D0 = free ear (before HA activation); D0 HA-on = on D0 and 30 min after HA are switched on; D + 30 HA-on = on D + 30 when HA are switched on; D + 30 HA-off = on D + 30 and 5 min after HA are switched off.

The Figure 3A shows the individual and average score obtained from the VAS-Loudness during follow-up and Figure 3B shows VAS-Loudness score differences (from D0) over time. The VAS-Loudness average scores decreased significantly between D0 HA-off and D + 30 HA-on (D0: 6.6 ± 1.4, D + 30 HA-on: 2.2 ± 1.7, Wilcoxon signed-test, *p* < 0.001, effect size of Cohen’s *dz* = 2). The statistics are summarized in Table 2. At D0, our results suggest that simply turning the HA on reduces tinnitus loudness. This is likely due to the masking effect produced by the HA. In line with this, there was no difference between D0 HA-on and D + 30 HA-off. Finally, there was a significant further reduction of tinnitus loudness between D0 HA-on and D + 30 HA-on, suggesting that the amplification had some sort of cumulative effects on tinnitus mechanisms: turning the HA on after 30 days of amplification provide more reduction of tinnitus intensity (mean VAS reduction of 4.4) than turning the HA on at baseline (mean VAS reduction of 3.1). Interestingly, the two SSD patients only reported a reduction of tinnitus loudness when the HA was turned on at baseline and at the end of the trial. There was no difference on tinnitus intensity between the loudness VAS when the HA was turned off between the beginning and the end of the trial (D0: 5.9 vs. D + 30: 5.7). For the AHL patients however, there was a clear reduction of tinnitus intensity for the same period (D0 vs. D + 30) and condition (HA off) with 6.8 and 3.9 VAS score, for the baseline and end of the trial, respectively. These results suggests that bilateral amplification is more efficient than monaural stimulation and/or that reversing the mechanisms responsible for tinnitus generation may differ between bilateral hearing loss vs. unilateral hearing loss. Finally, for the 3 patients with bilateral tinnitus, the VAS-Loudness score differences between D0 and D + 30 was significantly less compared with the loudness score difference of the 11 patients reporting unilateral tinnitus (3.2 vs. 4.8 decrease on average, for the bilateral and unilateral patients, respectively). Interestingly, the improvement in the loudness VAS at D0 when the HA was turned on was significantly correlated with the THI improvement over one month, Spearman Rho, *r*(12) = 0.64, *p* = 0.014. This result suggests that the masking effect of the amplification during the fitting could help predict the long-term benefit of the amplification on the tinnitus burden.

**Table 2:** Summary of the statistical differences of the VAS-Loudness scores between the test sessions.

D0	D0 HA-on	D + 30 HA-on	D + 30 HA-off	
**m = 6.6 ± 1.4** *(4.2–9.1)*	**−3.1 ± 1.7** *rank p = 0.001sign p = 0.000*	**−4.4 ± 2.2** *rank p = 0.000sign p = 0.000*	**−2.5 ± 2.0** *rank p = 0.001sign p = 0.002*	**D0**
	**m = 3.5 ± 1.2** *(1.8–5.6)*	**−1.3 ± 1.8** *rank p = 0.024sign p = 0.013*	**+0.6 ± 2.7** *rank p = 0.346sign p = 0.424*	**D0 HA-on**
		**m = 2.2 ± 1.7** *(0.0–5.0)*	**+1.9 ± 2.5** *rank p = 0.011sign p = 0.003*	**D + 30 HA-on**
			**m = 4.1 ± 2.4** *(0.2–7.6)*	**D + 30 HA-off**

White boxes indicate average (m) ± standard deviation and range (−) of each step during follow-up. Gray boxes indicate average evolution of the scores between each step ± standard deviation and rank *p* value/sign *p* value (Wilcoxon test). D0 = free ear (before HA activation); D0 HA-on = on D0 and 30 min after HA are switched on; D + 30 HA-on = on D + 30 when HA are switched on; D + 30 HA-off = on D + 30 and 5 min after HA are switched off.

### Usage of the different programs

**Figure 4:**
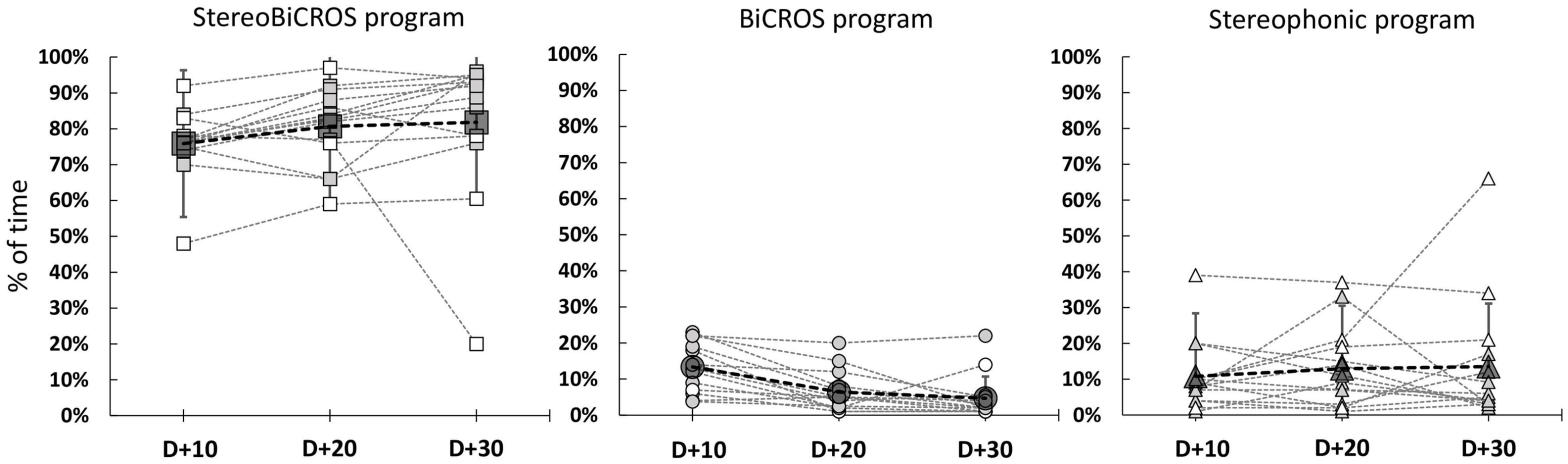
Evolution of programs usage during follow-up. Evolution of program usage during the 1-month trial period (D + 10 = 10 days after HAs activation, D + 20 = 20 days after activation and D + 30 = 30 days after HAs activation) recorded by datalogging. Gray dashed lines represent individual patients, gray symbols for patients with bothersome tinnitus (THI score > 38, *N* = 10) and white symbols for patients with non-bothersome tinnitus (THI score < 38, *N* = 4). Bigs symbols represent the mean ± SD at different times of follow-up.

**Figure 5:**
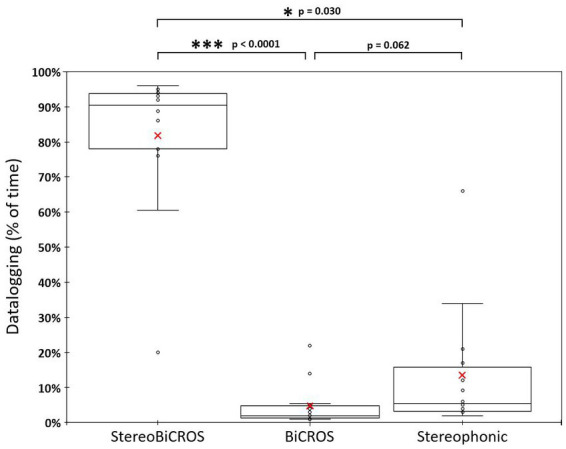
Distribution of program usage at D + 30. Distribution of program usage at D + 30 recorded by datalogging. Box and whisker charts show: minimum values, 1st quartile, median (horizontal bar), mean (red cross), 3rd quartile and maximum values. The StereoBiCROS program was the most used program (81.8% ± 20.5%), followed by the Stereophonic program (13.5% ± 17.6%) and the BiCROS program (4.7% ± 6.0%). *p*-values were obtained with Nemenyi’s test (pairwise multiple comparisons test) between each program.

HAs were worn regularly during the 1-month trial period to reach at D + 30 the mean daily usage duration of 12.6 ± 1.6 h per day, ranging from 10.0 to 14.6 h per day. Figure 4 allows us to follow the evolution of the use of each of the programs over time, and to verify that all the programs have been tested and used by the patients during the 1-month trial period (D + 10, D + 20 and D + 30). Figure 5 shows the final distribution of the program usage at D + 30. Datalogging shows that each program has been tried out and that the StereoBiCROS program was the most used program (81.8 ± 20.5%), followed by the Stereophonic program (13.5 ± 17.6%) and the BiCROS program (4.7 ± 6.0%). The difference in daily usage between the StereoBiCROS and the BiCROS programs was significant (Nemenyi’s test scored *p* < 10^−4^) as well as the difference between the StereoBiCROS and the Stereophonic programs (Nemenyi’s test scored *p* = 0.03). There was no significant daily usage difference between the BiCROS and the Stereophonic programs (Nemenyi’s test scored *p* = 0.06). By adding the stimulation duration of StereoBiCROS and Stereophonic programs, we can measure the total stimulation time of the poorer ear: it was stimulated 95.3 ± 5.9% of the time.

## Discussion

### Summary of the results

The aim of the present study was to investigate the effects of amplification of the poorer ear on tinnitus patients with AHL/SSD. A significant reduction in tinnitus loudness after 1 month of HA use was found, which persists when the device is turned off. An immediate decrease in tinnitus loudness was recorded after turning the HA device on, which was not related to the improvement in hearing levels. Finally, the tinnitus handicap was significantly improved during the trial period.

### Effects of HA amplification on tinnitus

Many studies over the past years have been carried out to investigate the effects of acoustic stimulation on tinnitus. Acoustic stimulation to improve tinnitus symptoms comes in two forms, noise generators and HA amplification, which may be combined. The reason for using acoustic stimulation to manage tinnitus is intuitive: it should be possible to interfere with tinnitus, as it is an auditory percept, with an auditory stimulus. The first objective behind acoustic stimulation is masking: an auditory stimulus may be used to mask (partially or completely) tinnitus, similar to an acoustic stimulation masking another stimulus-induced auditory percept. The *sine qua non* condition for this approach to be efficient is that the masking stimulus should be better tolerated by the patient than the tinnitus. This approach is recommended in different methods of clinical management, from Tinnitus Retraining Therapy (TRT) to the Cognitive Behavioral Therapies (CBT) (Jastreboff, 2007; Cima et al., 2012, 2014; Tyler et al., 2012). It is also believed that acoustic stimulation can interfere with the pathophysiological mechanisms of tinnitus (Noreña and Farley, 2013). Briefly, if tinnitus results from central changes after the sensory deprivation caused by hearing loss, the partial restoration of sensory inputs may prevent or reverse the tinnitus-related central changes (Noreña and Eggermont, 2005, 2006; Schaette and Kempter, 2006).

Although this approach (acoustic stimulation) is very popular worldwide (Henry et al., 2008, 2017; Cima et al., 2020), and has been used for years (Saltzman and Ersner, 1947; Hazell and Wood, 1981; Surr et al., 1985), there is only weak evidence for it. Indeed, while many studies report some benefits after sound therapy most studies are not randomized control trials (RCTs) (Hobson et al., 2012; Hoare et al., 2014b; Sereda et al., 2018). Another difficulty when investigating any clinical approach on tinnitus is that tinnitus severity is very sensitive to counseling and reassurance. It is often a challenge to disentangle the nonspecific effects of any clinical management that is related to reassurance, for instance, from more specific effects produced by the tested clinical approach. In this context, it is unclear whether acoustic stimulation should be compared to no treatment (waiting list), to an “inactive” (placebo) acoustic stimulation or if different sound therapies should be compared. In the RCT study of Parazzini et al. (2011), all patients received extensive counseling and were randomly assigned to one of two groups: one was fitted with open ear HAs, and the other with sound generators. On average, after 3 months of treatment, THI score was reduced by 20 points (from 60 to 40) and tinnitus loudness by 1.5 points (from 7 to 5.5) in the two groups (Parazzini et al., 2011). The design of this study makes it impossible to conclude whether tinnitus improvement has been caused by counseling, the sound therapy or both. Regardless of the cause of the reduction in tinnitus, the effects reported are similar to our current study with one month of hearing aid use: THI scores decreased by 30 points (from 45 to 15) and tinnitus loudness by 2.5 points. A recent RCT reported that HAs with noise generators and extended-wear HAs are equally good at alleviating tinnitus, indicated by a decline in the tinnitus functional index (from 55 to 30 points) (Henry et al., 2019). For comparison, a RCT study investigating the effects of CBT on tinnitus has shown an average improvement of 4.3 points (95% coincidence interval: [−7, −1]) after 3 months and − 7.5 after 12 months using the THI questionnaire (Cima et al., 2012). With effect sizes of Cohen’s *dz* = 1.5 and *dz* = 2, respectively, for THI and VAS-Loudness, results obtained in our study seem larger compared to those obtained after CBT, especially after only one month of treatment.

### The StereoBiCROS versus CROS devices

Overall, our study suggests that the effects of the StereoBiCROS is comparable or exceeding as more conventional hearing devices or validated approaches such as CBT in patients with different profiles of hearing loss. Hereby, the StereoBiCROS constitutes an interesting option for tinnitus patients with AHL/SSD. Before this system was available, patients with AHL/SSD were fitted essentially with CROS devices. These devices provide only unilateral auditory inputs by stimulating the better ear with the acoustic signal captured on the side of the poorer ear (Harford and Dodds, 1966). By favoring the good ear, the CROS devices contribute to increase the “contrast” between the good ear and the poor ear. One can speculate that by favoring the better ear and neglecting the poorer ear, the CROS devices may reduce the ability of the central auditory system to process auditory inputs through the poorer ear. In other words, the CROS devices may further increase the functional sensory deficit of the poorer ear. Assuming that tinnitus is the result of sensory deprivation, the CROS devices may then exacerbate tinnitus in the poorer ear. At the very least, the CROS devices may reduce the stimulation of the poorer (tinnitus) ear to interact with tinnitus through masking and residual inhibition. Therefore, tinnitus may become more salient and/or more difficult to put in the background. A study on bone-CROS devices (Faber et al., 2012) found out that 13 out of 14 patients with tinnitus observed no reduction of their tinnitus while using these devices. Authors specify that a bothersome tinnitus in the poorer ear might be a negative predictor for using CROS devices. Similarly in a recent study on 75 SSD/AHL patients fitted for 6 months with a CROS or BiCROS HA, the authors reported no improvement of tinnitus intensity or severity for the patients with tinnitus (Marx et al., 2021). This conclusion is supported by other studies (Desmet et al., 2012; Vincent et al., 2015; Van de Heyning et al., 2016) and the American Academy of Clinical Audiology’s Practice Guidelines (2015) which recommend that the device selection process includes considerations on both the presence and the severity of tinnitus on the poor ear.

In conclusion, by combining the BiCROS solution with the stimulation of the poor ear, the StereoBiCROS could provide a positive effect on tinnitus intensity and burden.

### Putative mechanisms accounting for tinnitus improvement

The underlying mechanism explaining the beneficial effect of StereoBiCROS on tinnitus remains unclear. A first mechanism is the masking of tinnitus produced by the acoustic amplification of the poorer ear. Tinnitus masking, whether it is partial or complete, by acoustic background may reduce patients’ awareness of their tinnitus (Folmer and Carroll, 2006; Trotter and Donaldson, 2008). In our study, the VAS-Loudness at D + 30 HA-on increases significantly 5 min after HA was switched off (D + 30 HA-off). This result suggests that part of tinnitus improvement results from acoustic masking. However, tinnitus loudness at D + 30 HA-off is significantly reduced compared to D0. This result is consistent with some long-lasting effects of acoustic stimulation on the tinnitus mechanisms. Tinnitus has been thought of as a consequence of the central plasticity triggered by sensory deprivation (Noreña, 2011). The partial bilateral restoration of the sensory inputs that were reduced by the hearing loss by the StereoBiCROS device may reverse the tinnitus-related central plasticity (Noreña and Farley, 2013). Interestingly, the effect of turning the hearing device on during the first fitting session was highly correlated with the THI improvement seen after one month, presumably due to masking. These results are in line with a previous study demonstrating a better long-term benefit of amplification in patients displaying partial or full masking of the tinnitus when the hearing aids are turned on than those displaying no to little masking (McNeill et al., 2012). Considering that traditional masking using a noise generator is not considered a suitable option in the case of severe to profound hearing loss, the current results suggest that masking through amplification is a suitable solution.

### Cochlear implants versus hearing devices and recommendations

Currently, CI is considered a standard effective treatment to bilateral severe-to-profound sensorineural deafness and an effective method to improve tinnitus condition (Baguley and Atlas, 2007; Andersson et al., 2009; Pan et al., 2009; Amoodi et al., 2011; Bovo et al., 2011; Olze et al., 2011; Kompis et al., 2012; Kim et al., 2013, 2016; Ramakers et al., 2015; Peter et al., 2019; Levy et al., 2020). However, it is necessary to consider both the cost/effectiveness ratio and the cost/risk ratio. The estimated cost of a CI (approximately € 25,000, Molinier et al., 2009) is much more expensive than that of a bilateral HA (usually between 2000 and 3,500 €). CI’s irreversibility following the possible loss of any residual hearing, surgical risks, and patients’ commitment to being involved in a rehabilitation program are all elements that must be taken into consideration.

The tinnitus improvement observed in the present study using StereoBiCROS is comparable to the effects produced by CI. Compared to CI, the StereoBiCROS only restores a part of binaurality but could still be a promising alternative as a tinnitus treatment for patients with AHL/SSD. This new stimulation appears to be far less destructive than CI and, in this context, we recommend the StereoBiCROS stimulation trial for a minimum period of 30 days with AHL/SSD patients, before considering cochlear implantation.

### Limitations of the study

Some caution is required when interpreting the results of the current study, because of the relatively small number of patients. Indeed, the sample’s size compromises the visibility of positive effects of the treatment and increases the possibility of false-negative results. It is well known that a lot of bias and placebo effects can occur in tinnitus studies or in treatment trials (Duckert and Rees, 1984; Dobie, 1999). Such effects could have partly influenced our results. Indeed, although precautions were taken when informing patients of the expected results of StereoBiCROS stimulation, potential biases may have occurred.

Besides, the pre-post study design cannot indicate whether it is the StereoBiCROS program which is responsible for the benefit, the other programs or all the programs. However, since almost all participants used the HA on average 12 h per day and mostly used the StereoBiCROS program, we are convinced that the poorer ear was stimulated continuously during the trial. This first study on StereoBiCROS stimulation is a technical pre-validation of a promising system but future studies should include more patients, a control group or a crossover method, a randomization of the different programs and a longer trial time to confirm our results.

## Conclusion

To our knowledge, the present study is the first to assess the effects of poorer ear amplification for the treatment of tinnitus in AHL/SSD patients using the StereoBiCROS device. This new solution appears as a credible alternative to CI in a population suffering from AHL/SSD associated with bothersome tinnitus. Therefore, we believe that the trial of a StereoBiCROS device should be included in the assessment before suggesting a CI. A minimum period of 30 days seems mandatory to assess the possible benefit for any patient suffering from AHL/SSD with disabling tinnitus.

## Data availability statement

The original contributions presented in the study are included in the article/supplementary material, further inquiries can be directed to the corresponding author.

## Ethics statement

The studies involving human participants were reviewed and approved by West 6 Personal Protection Committee N° 11,555-DM2. The patients/participants provided their written informed consent to participate in this study. Written informed consent was obtained from the individual(s) for the publication of any potentially identifiable images or data included in this article.

## Author contributions

All authors listed have made a substantial, direct, and intellectual contribution to the work and approved it for publication.

## Conflict of interest

MP is the owner of Laboratoire d’Audiologie Narbonne.

The remaining authors declare that the research was conducted in the absence of any commercial or financial relationships that could be construed as a potential conflict of interest.

## Publisher’s note

All claims expressed in this article are solely those of the authors and do not necessarily represent those of their affiliated organizations, or those of the publisher, the editors and the reviewers. Any product that may be evaluated in this article, or claim that may be made by its manufacturer, is not guaranteed or endorsed by the publisher.
